# Spatiotemporal characteristics of high-density gas jet and absolute determination of size and density of gas clusters

**DOI:** 10.1038/s41598-020-69824-z

**Published:** 2020-07-31

**Authors:** Bo Ram Lee, Prashant Kumar Singh, Yong Joo Rhee, Chang Hee Nam

**Affiliations:** 1 Center for Relativistic Laser Science, Institute for Basic Science, Gwangju, 61005 Republic of Korea; 2 Department of Physics, Gwangju Institute of Science and Technology, Gwangju, 61005 Republic of Korea

**Keywords:** Plasma physics, Techniques and instrumentation

## Abstract

Properties of gas clusters such as the size and number density when expanding into the vacuum after passing through a conical nozzle are analyzed for argon at an average density of 10^20^/cm^3^. Temporally and spatially resolved size and density distribution were measured from all-optical methods of Rayleigh scattering measurement and Nomarski interferometry using a CW laser. At the gas backing pressure of 80 bar, Ar clusters as large as 100 nm were obtained, which differs significantly from the size estimated by the conventional Hagena scaling law. The two independent methods of cluster characterization presented here would be useful to precisely determine the initial conditions in a variety of intense laser-cluster interaction driven applications such as neutron generation, thermonuclear fusion, efficient x-ray emission, and energetic ion acceleration.

## Introduction

The interaction of clusters with intense short-pulse lasers have played a crucial role in the success of various novel application such as high-temperature plasma production^1^, generation of fast electrons and ions via Coulomb explosion^2^, conversion of highly charged ions into neutral atoms^3^, x-ray generation^4,5^, phase-matching for nonlinear optics applications^6^, and nuclear fusion^7,8^. The atomic clusters, being an efficient absorber of the laser light, transfer a significant fraction of the laser energy to the kinetic energy of highly-charged ions via collisional heating of electrons. Thus, solid-density clusters, of size 10–1,000 nm, have emerged as an alternative targets for a compact, table-top, laser-driven energetic particle or photon sources^1,4,5,8-10^. However, for a clear understanding of the laser-cluster coupling mechanism and dynamics of the generated hot dense plasma, absolute characterization of the cluster parameters, such as size and density, becomes very crucial. Several methods have been used to determine the average size and density of the clusters such as molecular beam “slow-down”^11^, high-energy electron diffraction^12^, and time-of-flight mass spectroscopy^13^. The drawback of these methods is their sophisticated setup and the possibility of causing a severe disturbance in the cluster distribution during the measurement^13^. On the other hand, Rayleigh scattering is a non-disruptive technique widely used to estimate the cluster size^10,14–16^. However, the conventional Rayleigh scattering method alone does not provide sufficient information on the cluster properties. For instance, the cluster parameters are integrated over space and time and they are not quantified in absolute term. Therefore, a rigorous tool to characterize the cluster parameters is required in precise determination of the initial conditions during laser-cluster interaction experiments.

To overcome the limitation of Rayleigh scattering method, interferometry, as an additional diagnostics, can be used for estimating the absolute size and density of the clusters^15,17^. Here, by using these two independent diagnostics, we have obtained an absolute spatio-temporal map of argon cluster size and number density distribution. The difference of the present method from the earlier works^10,15,18^ lies in two aspects: (i) a nanosecond pulsed laser is replaced with a continuous wave (CW) diode laser (635 nm) of a few mW power. The temporal evolution of the gas expansion and the dynamics of cluster formation can be scanned by varying the exposure and the trigger time of CCD cameras. (ii) An expanded laser beam is used to obtain 2-dimensional (2D) Rayleigh scattering image of a cluster, instead of a 1D laser line focus scan method. Our 2D measurements show that the cluster size increases from few tens of nm to larger than 100 nm as one moves away from the nozzle along its central axis, while the cluster number density decreases accordingly. The nonlinear pressure dependence of the cluster size measured in the 2D Rayleigh scattering experiment, combined with the interferometry, coincides with the results estimated using the conventional 1D Rayleigh scattering method. A quantitative comparison of the measured results with the semi-empirical Hagena scaling law revealed a significant deviation. The method presented here provides necessary information to determine the initial conditions for future experiments where interaction of cluster with ultra-intense laser pulse will be studied. In the next section, two methods to determine the average size and number density of clusters are introduced followed by the discussion on generated argon clusters.

## Rayleigh scattering to diagnose the onset of cluster formation

Clusters can be produced through a supersonic adiabatic expansion of gas with high Hagena parameter into the vacuum through a nozzle, where the collisional mean free path is much smaller than the nozzle outlet size^10,13^. Atoms or molecules, mediated by the van der Walls force, undergo into the nucleation phase and achieve a quasi-equilibrium state. A cluster ensemble, consisting of 10^3^ to 10^7^ atoms, can be of the size of 10–100′s of nanometers^7^. The cluster size largely depends on the species of gas, temperature, backing pressure, and the nozzle geometry. The property of cluster can be described by a semi-empirical Hagena scaling law for axisymmetric gas expansion^19,20^:

1 $${n}_{c}=a{{\Gamma }^{*}}^{b}$$

where *n*_*c*_ is the number of particles in a single cluster, *a* and *b* are determined experimentally (Table 1). The semi-empirical Hagena parameter ${\Gamma }^{*}$ is defined as

2 $${\Gamma }^{*}={k}_{H}\frac{{d}^{0.85}{p}_{0}}{{T}^{2.29}}$$

with *k*_*H*_, the gas specific constant (1,650 for argon and 3.85 for helium^22^), *d,* the orifice diameter in µm, *p*_*0*_, the gas backing pressure in bar, and *T*, the gas temperature in Kelvin.

Since this scaling law was obtained from an experiment using sonic nozzles with low backing pressure^19^, the size of clusters produced from supersonic conical nozzles of a small opening angle at high backing pressure is usually overestimated. The effect of inner boundary layers in the conical nozzle is not taken into account^23,24^. In the case of a conical nozzle with a jet expansion half-angle of *δ*, *d* should be replaced with the equivalent diameter *d*_*eq*_ = *0.74d/*tan(*δ*). In the past decades, several experiments were performed to determine the constants *a* and *b* to match the Hagena scaling law in the given interval of Γ^*^ (Table 1). As this semi-empirical law was derived without detail consideration of parameters such as the heat from condensation, any additional constraints of flows^14,19,25^, or boundary layer effects^23^, the real cluster size and density may largely deviate from the calculated results. Moreover, the scaling law does not give any information about the expansion dynamics as well as the distribution of the gas, which would also affect the cluster growth rate^26^.

**Figure 1:**
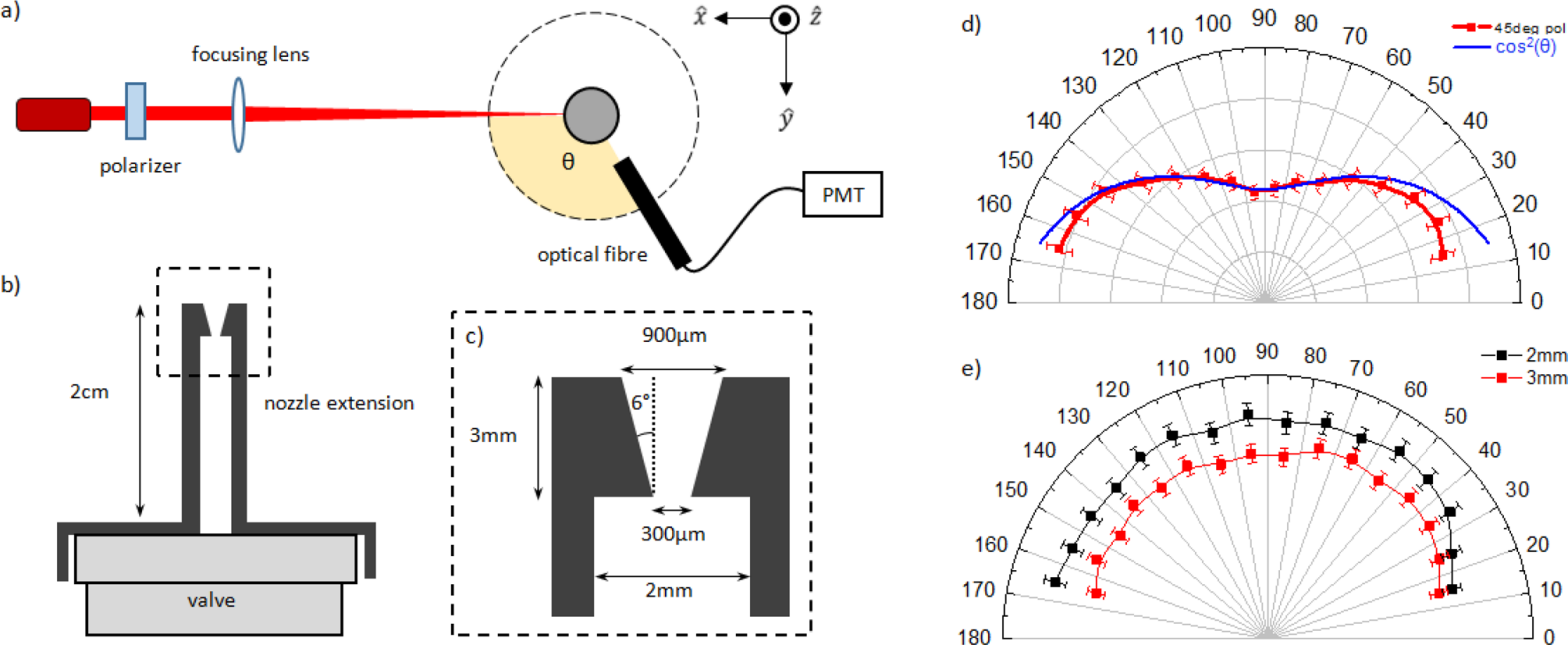
(**a**) Top view of Rayleigh scattering experimental setup. Linear polarized laser beam is loosely focused on a gas target. The scattered light is recorded via an optical fiber (Φ = 600 µm) located close to the target (2 cm), which is connected to a photomultiplier tube to increase its signal gain. (**b**) An extension is mounted on top of the gas valve to avoid the tightly focused beam to be partially blocked by a massive valve body close to gas outlet. (**c**) A conical throat at the extension tip shapes the gas jet profile of preferred geometry. (**d**) and (**e**) show the angular distribution of the scattered light signal along the scattered angle θ collected with an optical fiber. d) In case of the incident beam polarized 45 degree (along $\widehat{y}+\widehat{z}$), a cosine square relation is observed (red-measurement, blue-calculation). (**f**) The angular distribution for $\widehat{z}$-polarized laser beam is uniform in space as the measurement also shows. The decreasing intensity for increasing height from nozzle (from 2 to 3 mm) indicates that the number of scattering particles falls with height.

The experimental setup to prove the existence of clusters and to detect the angular distribution of Rayleigh scattered light is shown in Fig. 1a. A CW diode laser (wavelength: 635 nm; power: 3mW) was loosely focused on the gas jet down to 500 µm spot using a lens of 1 m focal length. The polarization axis of the diode laser was selected by a Glan-Taylor polarizer. A gas jet with a valve (Parker Hennifin series 9), assembled with a home-made nozzle extension tube (Fig. 1b,c), puffed argon gas in a vacuum chamber (~ 10^–3^ torr) with the backing pressure ranging from 40 to 80 bar in pulsed mode. The scattered light was collected using an optical fiber (Ocean optics, Φ = 600 µm, NA = 0.22), placed 2 cm away from the gas nozzle. The fiber was mounted on a motorized rotation stage to record the signal at different angles (*θ*) with respect to the laser propagation direction. The scattered signal collected by the fiber was amplified using a photomultiplier tube (Hamamatsu C123497) and read by a digital oscilloscope.

The angular dependence of Rayleigh scattering signal (*S*_*Rayleigh*_), if measured in the plane of incident beam polarization, ($\widehat{x}\widehat{y}$-plane, Fig. 1a), can be given as ^27^:

3 $${S}_{Rayleigh}\propto \frac{{I}_{0}}{{\lambda }_{L}^{4}}\left(1+{cos}^{2}\theta \right)$$

where *I*_*0*_is the incident beam intensity and *λ*_*L*_ is the laser wavelength. The angular dependence of the scattered light recorded with a beam, linearly polarized at 45 degree with respect to $\widehat{z}$ in the $\widehat{y}\widehat{z}$-plane (Fig. 1d), is compared with cos^*2*^*θ* function (blue curve). If the polarization axis of the beam is perpendicular to the observation plane, the angular distribution of the scattered light should be uniform. This was experimentally verified with the laser beam polarized along the $\widehat{z}$-axis (Fig. 1e). All measurements shown in this paper were performed with the laser beam polarized along the $\widehat{z}$-axis and the scattering signal was taken at θ = 90 deg, unless mentioned otherwise.

**Figure 2:**
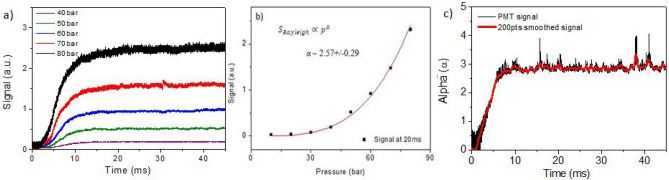
(**a**) Signal recorded at different backing pressure ranging from 40 to 80 bar over the gas valve opening time of 50 ms. Weak signals, taken at pressures lower than 40 bar are not shown. (**b**) The signal at t = 20 ms taken at different gas backing pressure was fitted by a power-law $\propto$ p^α^. α is estimated to be 2.57 $\pm$ 0.29. (**c**) To obtain the timely change of α, an exponential fitting was performed for the signal as a function of gas backing pressure at each recorded time interval of ∆t = 1 µs (here, data taken at pressures lower than 40 bar are taken into account). The exponent α derived from this nonlinear relation (black) changes with time and finally arrives in a stabilized state from 15 to 50 ms, α being 2.8 $\pm$ 0.2. The red line shows a smoothed signal trend over 200 points. For all measurement, the data has been averaged over 3 shots with its error of less than 5%. The time resolution is ∆t = 1 µs.

A typical temporal evolution of the measured Rayleigh scattering signal is shown in Fig. 2a). After the gas starts to flow into the chamber at t = 0, the valve remains open for different durations varying from 5 to 60 ms. During the valve opening duration larger than 10 ms, the Rayleigh scattering signal is almost constant and disappears within 5 ms of closing the valve. The measurement shows that finite time is needed for the scattered signal to reach the saturation stage. Previous works indicate that the saturation time scale can vary depending on the types of nozzle and solenoid valve used due to the geometrically different expansion of the gas^10,18^. The saturation phase was not observed for short valve opening time, such as for 5 ms. Therefore, for a stable laser cluster interaction, the valve opening time should be long enough to reach the saturation stage.

The nonlinear increase of Rayleigh scattering signal with backing pressure is useful for determining the onset of clustering according to the relation^18,26^:

4 $${S}_{Rayleigh}\propto {N}_{0}{n}_{c}\propto {N}_{0}{p}^{b}\propto {p}^{b+1}\propto {p}^{\alpha }$$

where *N*_*0*_ is the number density of neutral gas, *n*_*c*_, the number of atoms in a cluster, *p*, the gas backing pressure. From the known relations, ${N}_{0}\propto p$ and ${n}_{c}\propto {p}^{b}$, the dependence of *S*_*Rayleigh*_ on *p* can be simply denoted as *S*_*Rayleigh*_ $\propto {p}^{\alpha }$ whereas *b* and *α* are to be determined experimentally. The exponents in Eq. 4 can be derived from the power-law fitting to the scattering signals obtained at different gas backing pressures (Fig. 2b). To best of our knowledge, no previous work has shown the temporal evolution of parameter *α*, but instead, a steady value of *α* was obtained. Figure 2c) visualizes the temporal evolution of *α*, which shows the time-dependence change of cluster characteristics. The parameter *α* at first increases linearly up to 12 ms, then remains constant at the value of 2.8 $\pm$ 0.2. The error comes from the smoothing of signal by 1,000 points and is calculated to be about less than 10%. In the present experiment, Hagena parameter Γ^*^ ranges from 6.2 $\times$ 10^4^ to 5 $\times$ 10^5^ in the interval of 10 to 80 bar at room temperature (*T*_*0*_ = 295.15 K) with the given nozzle geometry (*α* = 6 deg, *d* = 900 µm) as shown in Fig. 1c). In this range of Γ^*^, previous experimental works on cluster size determination^12-14^ suggest that the exponent *b* of the Eq. (1) is to be 1.8 (Table 1), giving *α* = 2.8 (Eq. 4), in agreement with our measurement in Fig. 2c). From this relation, one can infer that *n*_*c*_ turns out to be in a range from 10^5^ to 10^6^ per cluster for the pressure ranging from 10 to 80 bar. Accordingly, the average cluster size (*a*) can be estimated from the number density of a cluster *n*_*c*_, as $a\approx 0.1\times \sqrt[3]{9{n}_{c}}$ nm as in ^16^. In our experimental condition, average size of the cluster is expected to lie in the interval of 10 nm to 40 nm for the given backing pressure range from 10 to 80 bar. As the cluster size (~ 10 nm) is much smaller than the wavelength of diode laser (635 nm), we are indeed in the Rayleigh scattering regime as confirmed from our measurements (Fig. 1d,e).

## Scattering side image combined with interferometry

For absolute cluster size determination, the Rayleigh scattering diagnostic alone does not suffice as both, the density and the size of clusters, are unknown. With this method, only a rough estimate of cluster size can be determined, as described in the previous section. For an absolute cluster size calibration, Nomarski interferometry is employed in addition to the Rayleigh scattering method. By employing these two independent diagnostics, the spatial distribution of the average size and number of clusters per unit volume can be calculated as introduced by Kim et al.^15^ in their 1-dimensional measurement. The present work shows a temporally and spatially resolved 2-dimensional map of number density and size of clusters.

**Figure 3:**
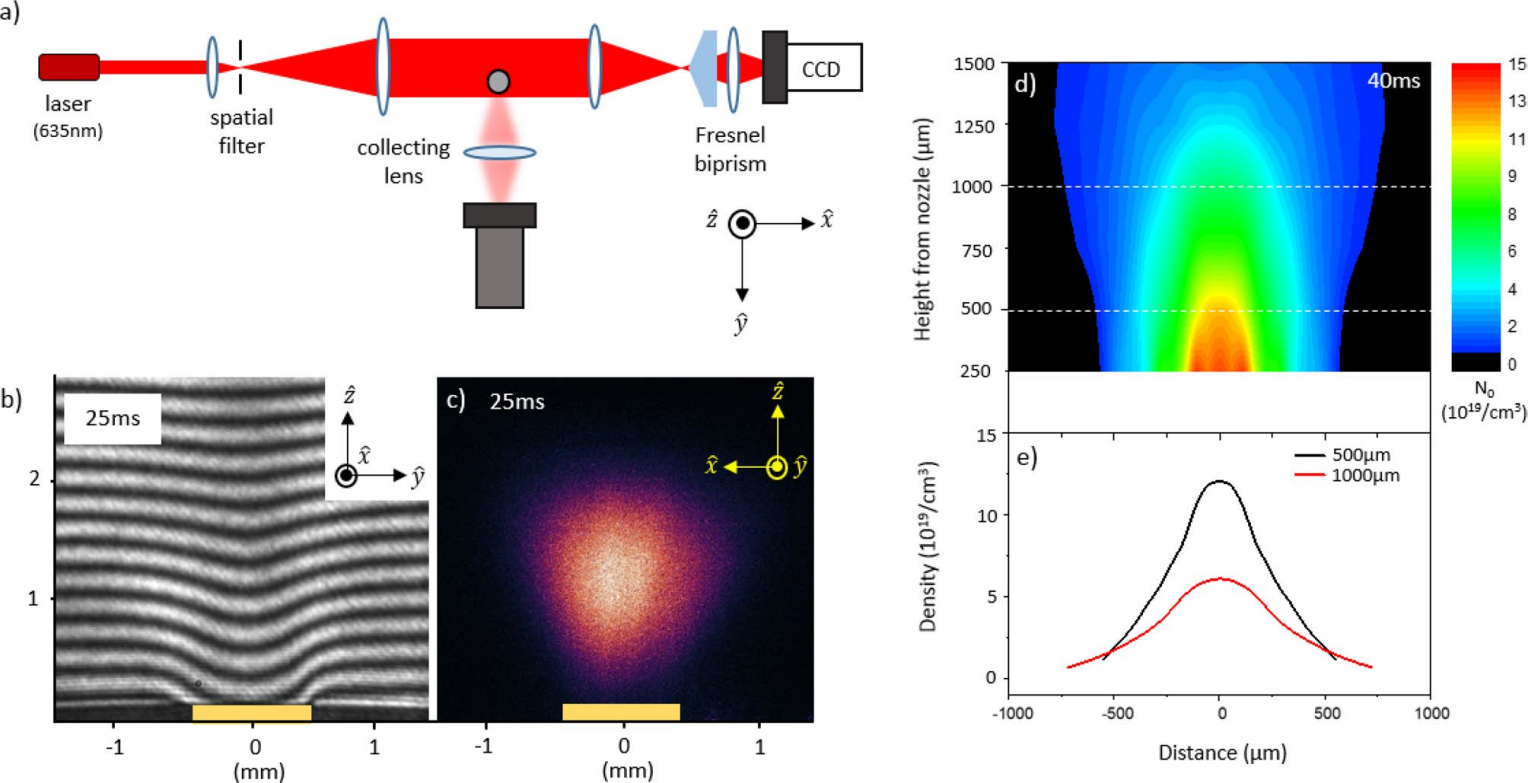
(**a**) Top view of the experimental setup where the 2-dimensional side scattering imaging is combined with interferometry. (**b**) Raw data of interferometry with clear fringe shift due to dense argon gas jet at 80 bar, and (**c**) side scattering image are shown. These images are taken at 25 ms after opening the gas valve with integration over 5 ms. The gas valve was opened for 70 ms. The yellow rectangle on the bottom indicates the outlet of nozzle (Φ = 900 µm). (**d**) Contour plot of the spatial density distribution of argon gas jet at 80 bar, about 40 ms after the valve opening. Sideway areas of low gas density are masked in black. (**e**) Density profiles at two different heights from the nozzle, 500 µm and 1000 µm (white dashed lines in** d**)), are plotted in black and red, respectively. It shows that the density near nozzle tip slightly exceeds 10^20^/cm^3^. For this measurement, the valve is kept open for 70 ms.

The Nomarski interferometry setup is placed along the laser propagation direction (Fig. 3a). The expansion of the laser beam diameter ensures that only half part of the beam passes through the gas jet region and the other unperturbed part serves as a reference. The phase difference experienced by the beam travelling through the gas medium is recorded as a fringe shift in the interferogram (Fig. 3b). For the conical nozzle used in our setup, the medium is expected to be cylindrically symmetric. Assuming this symmetry, the radial density distribution from the phase shift measurement is deduced after performing the Abel transformation^28^ using IDEA software^29^ Fig. 3d) shows a 2-dimensional neutral gas density distribution recorded 40 ms after the opening of valve. The generated gas jet density exceeds the level of 10^20^/cm^3^ near the nozzle outlet and falls gradually as moving away from the nozzle. In our experimental set up, it was not possible to obtain a clear phase map for the gas density lower than 10^18^/cm^3^. These unresolved areas are masked in black to avoid any ambiguity. The lineouts of the transverse gas profile at two different heights, 500 µm (black) and 1000 µm (red) are shown in Fig. 3e). For a clustered gas, the intra-cluster density *n*_*c*_ might be orders of magnitude higher than the inter-cluster density *N*_*c*_. The interferogram merely provides an average density *N*_*0*_ information. Therefore, we have combined the 2-dimensional Rayleigh scattering measurements with the 2-dimensional interferogram to obtain the absolute size and density of clusters in the gas medium.

**Figure 4:**
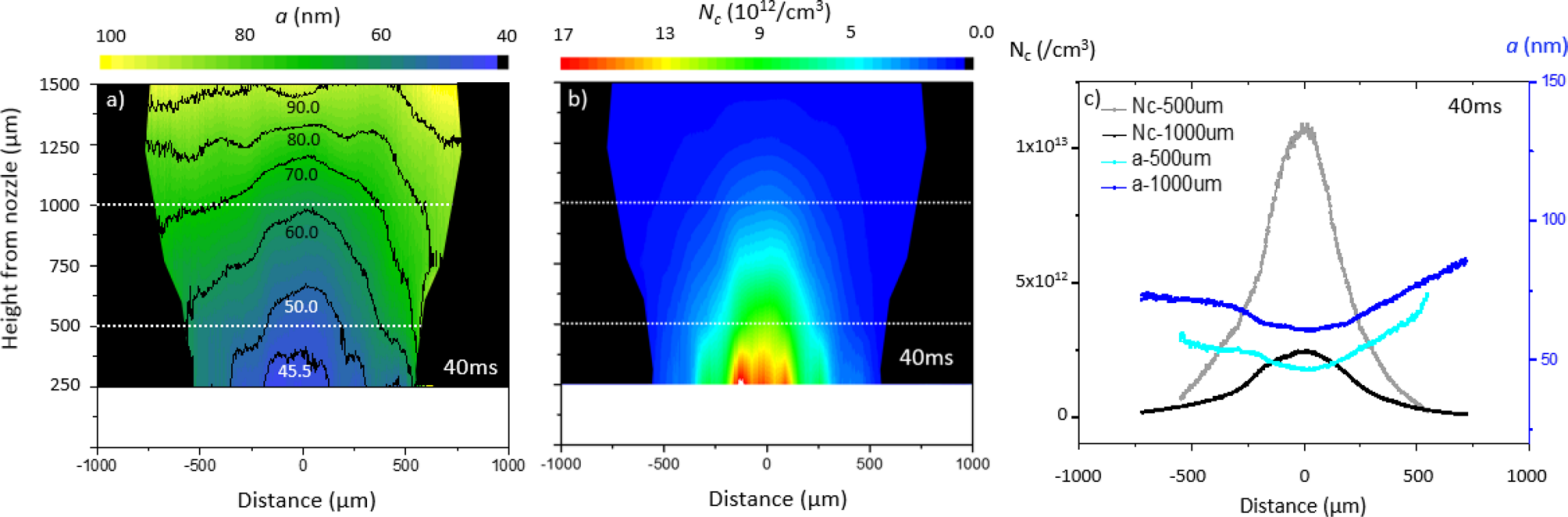
(**a**) Spatial distribution of average argon cluster size a and (**b**) argon cluster density N_c._ Black area corresponds to region which is not analyzable for interferometry due to low gas density, as in Fig. 3d. (**c**) The cluster density (gray at 500 µm and black line at 1000 µm) and the size of clusters (light blue at 500 µm and blue at 1000 µm) at different heights are shown. Measurement was done at gas backing pressure of 80 bar with CCD exposure time of 5 ms and at 40 ms after the gas valve was opened.

For the 2-dimensional measurement of Rayleigh scattering signal, the laser beam was expanded to a diameter of 1.5 cm, large enough to cover most of the expanded gas region of ~ 5 mm. The imaging system facilitates a 2D space-resolved Rayleigh scattering signal in contrast to the 1D measurements performed with an optical fiber. By varying the exposure and triggering time of CCD, the temporal evolution of cluster formation was obtained. The raw data of the scattered side image (Fig. 3c) carries an imprint of the laser beam profile. While analyzing the scattered signal, non-uniformity in the transverse profile of the incident laser beam was considered. This step was useful in the accurate determination of the cluster parameters such as the average cluster size $\stackrel{-}{a}$ and inter-cluster density *N*_*c*_. These parameters can be calculated using the following Eqs. ^15,17^:

5 $$\stackrel{-}{{a}^{6}}{N}_{c}=\frac{1}{\pi {k}^{4}}{\left|\frac{\varepsilon +2}{\varepsilon -1}\right|}^{2}\frac{\Delta {E}_{lens}}{{E}_{in}\Delta x}\frac{1}{\left({\beta }^{2}-{\beta }^{4}/4\right)}$$

6 $$\stackrel{-}{{a}^{3}}{N}_{c}=\frac{\Delta {n}_{r}(x)}{2\pi }\left(\frac{\varepsilon +2}{\varepsilon -1}\right)$$

where the Eq. (5) describes the factors depending on the geometrical light collection set up and the Eq. (6) shows the effect of the neutral gas density on the cluster size and the number density. In these equations, *k* is the wave number, *ε* the dielectric function of the bulk material internal to the cluster (*ε* = 1.67 for solid argon ^30^), *∆n*_*r*_ the radial refractive index shift caused by the gas, *E*_*in*_ the laser energy incident on the scattering volume, and *∆x* the propagating length of laser through the gas. *∆E*_*lens*_ is the scattered energy collected by the lens and its half-angle is defined as *β* = tan^-1^(*R*_*0*_/*h*)$\approx$ *R*_*0*_/*h* where *R*_*0*_the lens radius and *h* the distance between the lens and scattering volume. Combining these two equations, the spatial distribution of the cluster radius *a* and inter-cluster density *N*_*c*_was obtained. Results are shown in Fig. 4. The black areas in Fig. 4a,b correspond to the region masked out in the interferogram (Fig. 3d). The cluster size map (Fig. 4a) shows that the cluster radius increases while moving away from the nozzle. For instance, the small ones with radius of ~ 45 nm reside near the nozzle, whereas large clusters with size of ~ 80 nm are located about 1 mm away from the nozzle. This suggests that as the gas expands and cools down, the particles start to stick together effectively and form larger clusters. In contrary, the inter-cluster density (${N}_{c}$) falls down while moving away from the nozzle. The reverse trend is caused by the gas jet expansion and clustering of the gas molecules (Fig. 4b). Line profile of the number density *N*_*c*_and the cluster radius *a* at two different heights from the nozzle, 500 µm and 1000 µm, are shown in Fig. 4c). A clear trend of decrease in cluster density (light gray and black) as well as increase in cluster size (light blue and blue) is observed as one moves further away from the nozzle. The growth of cluster size along the radial directions is also seen due to the transverse cooling of expanding gas (Fig. 4a).

**Figure 5:**
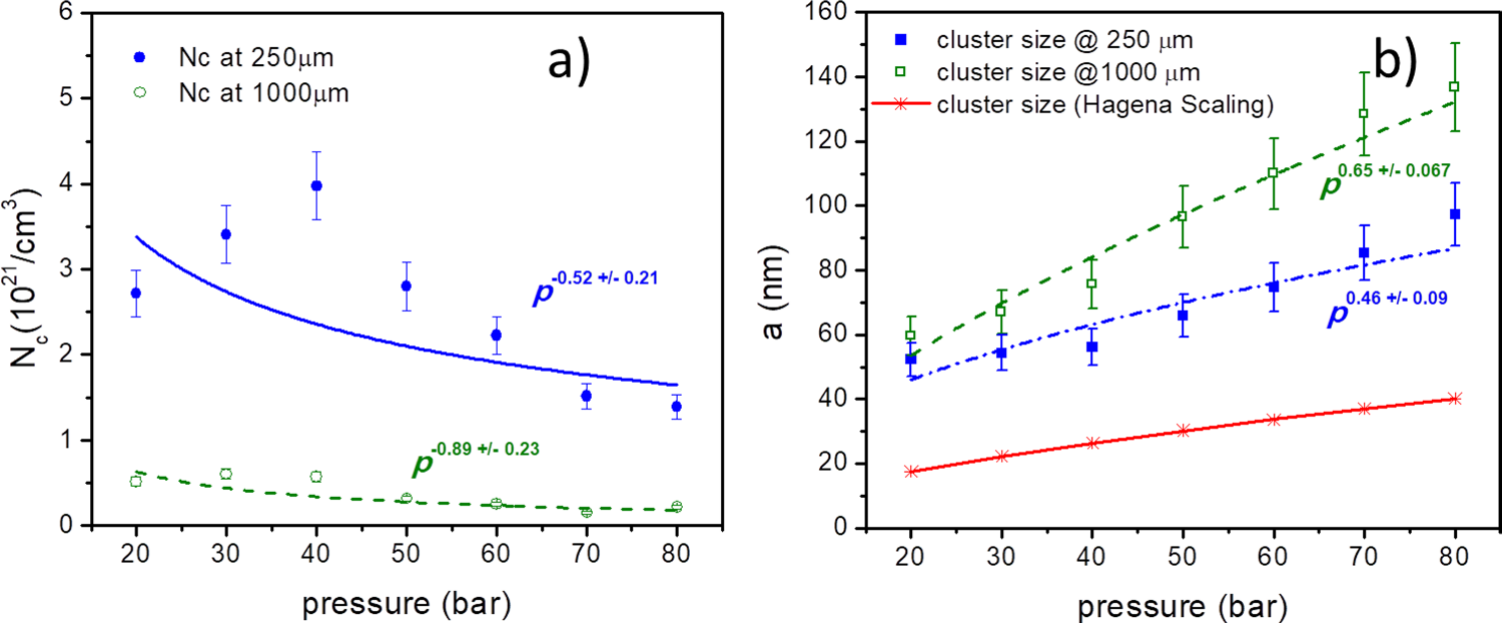
(**a**) Cluster density N_c_ and (**b**) cluster size a, shown for different backing pressures at 250 µm and 1000 µm height from nozzle along its central axis. For comparison, the calculated cluster size according to Hagena scaling law (**b**) is plotted in red. To obtain this data, CCD recorded the scattered light for 70 ms equal to the valve opening duration.

Furthermore, the dependence of the gas backing pressure *p* on the cluster density ${N}_{c}$ and cluster radius $a$ is shown in Fig. 5(a,b), respectively. The cluster density (Fig. 5a) falls down with pressure, whereas the cluster size (Fig. 5b) grows with the backing pressure. For example, at backing pressure of larger than 60 bar, the clusters of size larger than 100 nm can be found at the height of 1000 µm. The cluster densities *N*_*c*_ at pressure lower than 40 bar is expected to be larger than the measured data. The discrepancy might be caused by the large error/noise of the interferometry for neutral gas density measurement lower than 10^18^/cm^3^. From *S*_*Rayleigh*_ $\propto$ *N*_*0*_*n*_*c*_ $\propto$ *N*_*c*_*a*^*6*^ $\propto$ *p*^*α*^, the power index *α* is estimated from the nonlinear fitting of *a* and *N*_*c*_ to the gas backing pressure *p* (Fig. 5). At the height of 250 µm from the nozzle, the exponent *α* is found to be 2.24 $\pm$ 0.75, while at 1000 µm height, *α* is 3.01 $\pm$ 0.65. Interestingly, the average value of exponent *α* (~ 2.6) derived from the 2D measurement described above, is similar to the one estimated from the 1D-Rayleigh scattering method (*α* = 2.8, Fig. 2c). However, it should be emphasized that the 2D spatially resolved Rayleigh scattering measurement has a higher accuracy in the determination of cluster size and density compared to the conventional 1D method. Experimentally derived value of cluster size *a* (Fig. 5b) is compared against the result calculated by the Hagena scaling law (Fig. 5b). This semi-empirical scaling law while relying on the gas backing pressure does not contain any dependence on the local position of the clusters. The Hagena scaling law underestimates significantly the cluster size in the given experimental condition. In this work, the beam size was not large enough to cover the area where the cluster size is expected to level out while the cluster density decreases. It is left for the future work where a much broader picture of the cluster size distribution can be presented.

## Conclusion

In summary, we have presented a 2D spatial–temporal characterization of argon clusters formed during the expansion of a 10^20^/cm^3^ density gas jet. By combining results from two independent techniques, namely Rayleigh scattering and Nomarski interferometry, a 2D map of the absolute size and number density of cluster was obtained. Although the conventional 1D Rayleigh scattering data, collected by the optical fiber, showed reasonable agreement with a 2D imaging technique, the 2D method adopted here revealed a detailed picture of the clusterization process. At the gas backing pressure of 80 bar, clusters as large as 100 nm were obtained, which differs significantly from the size estimated by the conventional semi-empirical Hagena scaling law. Our analysis indicates that the Hagena’s empirical law, although suits well as a general guide to the cluster formation, but may lack sufficient accuracy in the cluster size characterization. We believe that our method of characterizing the cluster parameter would facilitate the precise determination of initial conditions in a variety of intense femtosecond laser-cluster interaction such as energetic neutron generation, thermonuclear fusion, x-ray emission, and energetic ion acceleration. The importance of having well-characterized cluster parameters can be highlighted from the ion acceleration during the Coulomb explosion of a cluster. The dependence of the maximum ion energy (*E*_*max*_) on cluster parameters is given as^31^:

7 $${E}_{max}\approx 300{z}^{2}\times {\left(\frac{{n}_{0}}{5\times {10}^{22}{cm}^{-3}}\right)}^{1/2}\left(\frac{{R}_{0}}{1 \mu m}\right)MeV (2)$$

where ${n}_{0}$ is the cluster density in cm^-3^, $z$ the ion charge state in cluster and ${R}_{0}$ the cluster radius in μm. Here, a precise knowledge of cluster density and size could be helpful in exact determination of the maximum ion energy achieved from the cluster explosion.

**Table 1:** Experimentally determined *a* and *b* for a different range of Hagena parameter ${\Gamma }^{*}$ expressed in Eq. (1).

${\boldsymbol{\Gamma }}^{\boldsymbol{*}}$ range	*a*	*b*	*n*_*c*_	Ref
350 < Γ* < 1,800	38.4	1.64	2.66 $\times$ 10^5^	^21^
1,000 < Γ* < 7,300	33	2.35	1.053 $\times$ 10^7^	^19,20^
2,100 < Γ* < 14,000	78	1.84	1.59 $\times$ 10^6^	^19^
10^4^ < Γ* < 10^6^	100	1.8	1.64 $\times$ 10^6^	^16^
